# A Semantic Labeling Approach for Accurate Weed Mapping of High Resolution UAV Imagery

**DOI:** 10.3390/s18072113

**Published:** 2018-07-01

**Authors:** Huasheng Huang, Yubin Lan, Jizhong Deng, Aqing Yang, Xiaoling Deng, Lei Zhang, Sheng Wen

**Affiliations:** 1 College of Engineering, South China Agricultural University, Wushan Road, Guangzhou 510642, China; huanghsheng@stu.scau.edu.cn (H.H.); ylan@scau.edu.cn (Y.L.); 2 National Center for International Collaboration Research on Precision Agricultural Aviation Pesticide Spraying Technology, Wushan Road, Guangzhou 510642, China; dengxl@scau.edu.cn (X.D.); zhanglei@scau.edu.cn (L.Z.); vincen@scau.edu.cn (S.W.); 3 College of Electronic Engineering, South China Agricultural University, Wushan Road, Guangzhou 516042, China; yangaqing@stu.scau.edu.cn; 4 College of Agriculture, South China Agricultural University, Wushan Road, Guangzhou 516042, China; 5 Engineering Fundamental Teaching and Training Center, South China Agricultural University, Wushan Road, Guangzhou 510642, China

**Keywords:** UAV, remote sensing, weed mapping, Deep Fully Convolutional Network, semantic labeling

## Abstract

Weed control is necessary in rice cultivation, but the excessive use of herbicide treatments has led to serious agronomic and environmental problems. Suitable site-specific weed management (SSWM) is a solution to address this problem while maintaining the rice production quality and quantity. In the context of SSWM, an accurate weed distribution map is needed to provide decision support information for herbicide treatment. UAV remote sensing offers an efficient and effective platform to monitor weeds thanks to its high spatial resolution. In this work, UAV imagery was captured in a rice field located in South China. A semantic labeling approach was adopted to generate the weed distribution maps of the UAV imagery. An ImageNet pre-trained CNN with residual framework was adapted in a fully convolutional form, and transferred to our dataset by fine-tuning. Atrous convolution was applied to extend the field of view of convolutional filters; the performance of multi-scale processing was evaluated; and a fully connected conditional random field (CRF) was applied after the CNN to further refine the spatial details. Finally, our approach was compared with the pixel-based-SVM and the classical FCN-8s. Experimental results demonstrated that our approach achieved the best performance in terms of accuracy. Especially for the detection of small weed patches in the imagery, our approach significantly outperformed other methods. The mean intersection over union (mean IU), overall accuracy, and Kappa coefficient of our method were 0.7751, 0.9445, and 0.9128, respectively. The experiments showed that our approach has high potential in accurate weed mapping of UAV imagery.

## 1. Introduction

Rice is the world’s most important crop. Currently, more than one third of the world’s population relies on rice as their principal food [1]. However, weed infestations present great challenges for rice cultivation. Weedy rice populations have been reported in many rice growing areas in the world, from rice transplanting to direct seeding [2]. The weeds compete with rice for light, water, and nutrients, which may cause serious yield losses [3]. Weed control in rice fields is necessary, but inappropriate herbicide treatment has led to agronomic and environmental problems [4]. Usually, herbicide treatments are applied at a constant dose by specific machinery (i.e., UAVs, tractors), ignoring the spatial distribution of weeds. Such operations may lead to excessive use of herbicide since somewhere it is needed less or it is not needed at all, increasing the risk for environmental pollution. To appropriately address this problem, it is necessary to integrate the weed control with Site-Specific Weed Management (SSWM). In the context of SSWM, the herbicide is only applied with the presence of weed infestation, and the dose should be adjusted according to weed densities.

However, before carrying out a SSWM task, an accurate weed distribution map is needed, which may provide decision support information for the spraying machinery [5]. Usually, the optimal time for most herbicide treatment is at the early growth stages (i.e., seedling or tillering stages) of weeds and rice. Thus, weed mapping in this period is significant in real applications. UAVs are able to fly at a low altitude [6], capturing imagery at a very high resolution [7], which is suitable for mapping weeds during their early growth stages. Though UAV remote sensing was proven to be effective in weed mapping tasks [8,9], the conversion of UAV imagery into accurate weed distribution maps is still the main bottleneck in SSWM applications.

Several studies have employed machine learning methods [8,9] for UAV imagery weed mapping tasks. Alexandridis et al. [8] applied four machine learning approaches to map the distribution of *S. marianum* in a field. The adopted architecture were One Class Support Vector Machine (OC-SVM), One Class Self-Organizing Maps (OC-SOM), Autoencoders and One Class Principal Component Analysis (OC-PCA). Experimental results demonstrated that the OC-SVM obtained best performance in *S. marianum* identification, and the overall accuracy was up to 96%. However, traditional machine learning approaches only involve low-level hand-engineered features (i.e., color or texture features) [10] for classification, which tend to be less precise [11] and hard to generalize.

Deep learning methods are automatic feature learning approaches which transform input data into representations at a higher and more abstract level [12]. As one of the classical architectures of deep learning, fully convolutional network (FCN) has achieved state-of-art performance in semantic labeling tasks in computer vision [13], which also shows great potential in remote sensing applications. Zhang et al. [14] presented a detailed investigation on the sensitivities and contributions of each layer in FCN, and built an optimal layer fusion architecture. Experiments were performed on two public datasets (ISPRS Vaihingen 2D Semantic Labeling dataset and Potsdam dataset), and impressive results were observed. Volpi et al. [15] presented a FCN-like network for dense semantic labeling task. The proposed network performed downsampling through convolutions and upsampled them to full resolution by deconvolutions. Experiments showed that the presented approach obtained results aligned with the state-of-art models on two challenging datasets (Vaihingen and Potsdam sub-decimeter resolution datasets). However, most of the previous studies mainly focused on the land cover classification tasks in remote sensing. To the best of our knowledge, related studies on weed mapping tasks in UAV imagery using semantic labeling approaches are not found, except for the previous work of our team [16]. In our previous work, the FCN framework was applied for weed mapping of UAV imagery. We adapted the ImageNet pre-trained VGG-16 net and transferred their learned representations to our semantic labeling task. Skip architecture was used to improve the prediction accuracy. Though FCN was proven to be effective in [16], the detection for small weed patches was still the bottleneck of generating an accurate weed distribution map. In this paper, we still follow the general idea of fully convolutional fashion. However, several new features are added to address the problems of previous work: (1) residual learning and deeper architecture are applied to further improve the accuracy; (2) a new type of convolution (atrous convolution) is adopted to extend the field of view of convolutional filters; (3) the impact of multi-scale processing (Atrous Spatial Pyramid Pooling) was evaluated; (4) the fully connected CRF was employed as the post processing to further refine the spatial details. All these issues will be addressed in the methodology section.

The objective of this work is to produce an accurate weed distribution map for the UAV imagery, and ultimately provide decision support information for herbicide treatment applications. The framework of this paper is arranged as follows: Section 2 introduces the process of collecting data, Section 3 shows the analyzed accurate weed mapping methodology, Section 4 presents the results and discussion, and Section 5 presents the conclusions and future work.

## 2. Data Collection

### 2.1. Study Site

**Figure 1:**
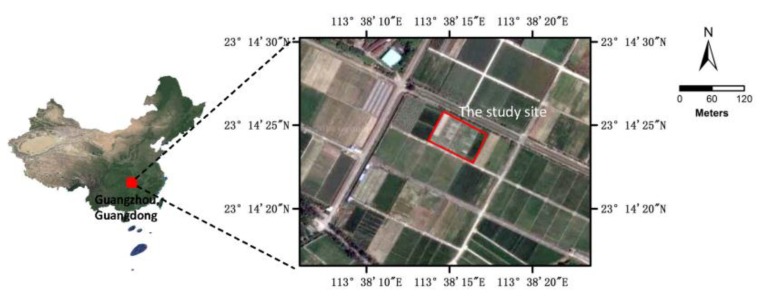
The general location of the study site.

**Figure 2:**
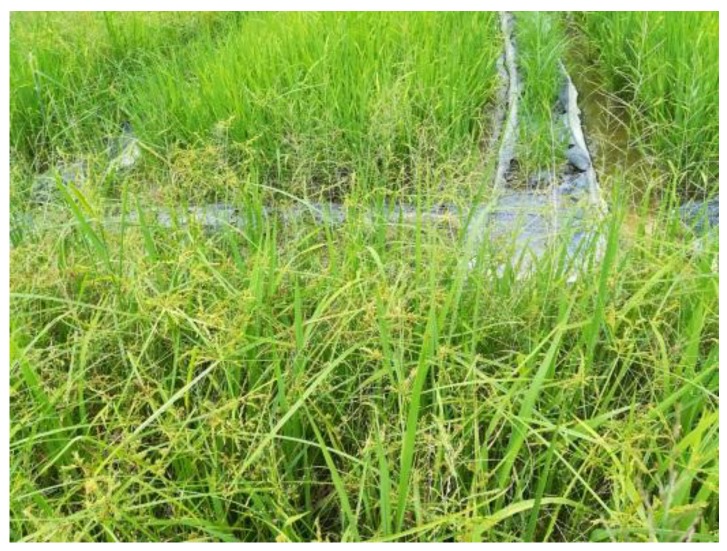
An overview of the weed patches in the field.

The study site was located in a rice field in South China (113.636888 N, 23.240441 E), as shown in Figure 1. The rice field had an area of 0.54 ha (90 × 60 m^2^) and the ground was flat. The field was plowed and sown with Huahang No. 31 [17] at a seeding rate of 60 kg·ha^−1^ on 21 August 2017, with a row spacing of 50 cm. N and P_2_O_5_ were applied at the dose of 40 kg·ha^−1^ and 50 kg·ha^−1^, respectively. No obvious presence of diseases or insect infestations was observed in this field during the growth stages. The rice field was naturally infested with *Chinese sprangletop* (*L. chinensis*) [18] and *Cyperus iric* [19], as shown in Figure 2. All these weeds can be treated with the same type of herbicide. The rice and weeds were both in the principal stage 2 (Early Tillering, 3–5 tillers detectable, codes 23–25) from the Biologische Bundesanstalt, Bundessortenamt und CHemische Industrie (BBCH) extended scale [20].

### 2.2. Data Collection

#### 2.2.1. Data Collection

**Figure 3:**
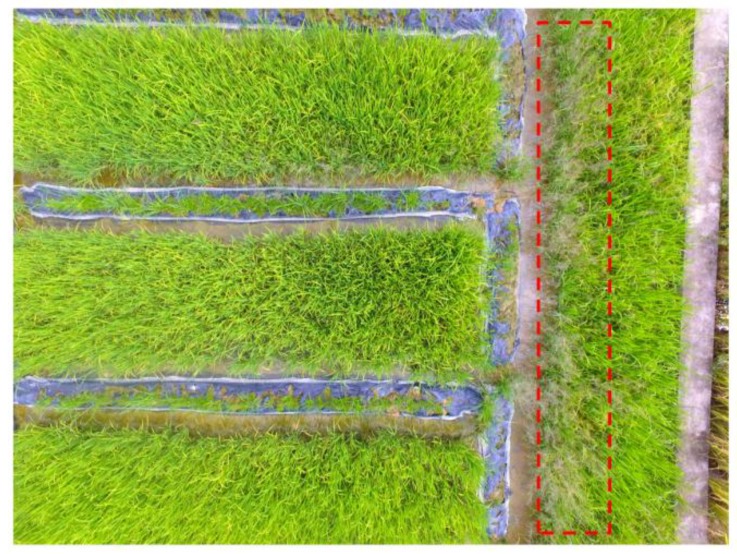
An example of the UAV imagery collected in the experiment.

UAV data was collected on 2 October 2018, when the weeds and crops were in their early tillering stages. Weed management (i.e., herbicide treatment) is usually recommended at this stage. A rectangle area of 90 × 60 m^2^ was delimited for UAV data collection, and a quad-copter UAV (Phantom 4, SZ DJI Technology Co., Ltd., Shenzhen, China) was used to perform the flights. The typical technical characteristics of the Phantom 4 are listed in Table 1. The coordinates of four corners were collected for automatic mission planning. The flight height was set to 6 m above the ground, and the side-lap and the end-lap of imagery were set to 50% and 60%, respectively. After that, the UAV was started to perform the flights and capture the imagery automatically, according to the planned mission. In this experiment, 91 UAV imagery were captured. Figure 3 gave an example of the collected imagery. From Figure 3 (the weed patches are indicated by red dashed lines) we can see that, the rice and weeds can be directly distinguished since the resolution (0.3 cm) was sufficient for visual discrimination.

#### 2.2.2. Dataset Description

**Figure 4:**
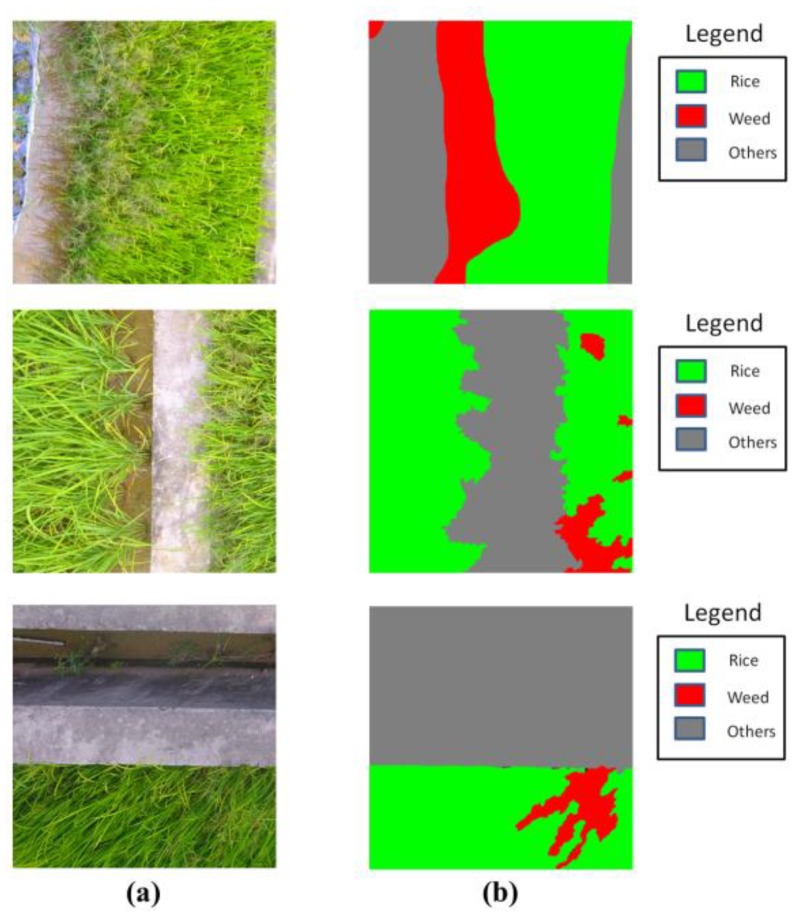
Three samples of our dataset: (**a**) aerial images; (**b**) corresponding GT labels.

In our work, ground truth (GT) maps with pixel correspondence were needed to evaluate the performance of the classifiers. We manually labeled the UAV imagery at the pixel level under the instruction of agronomic experts. The labeling was conducted by hand for the total of 91 UAV images, and labeling each image took 40 min on average. However, the resolution of the collected imagery was 4000 × 3000, making it a great challenge to train a deep neural network with limited GPU memory. In order to perform the training and inference on the UAV imagery at its original resolution, we followed the general idea of [21] and split each image into non-overlapped regions of 1000 × 1000. Thus, our dataset contained 1092 UAV images (size 1000 × 1000), with 1092 GT maps (size 1000 × 1000). For each UAV image, there existed a GT map having a pixel-level correspondence with it. Three image-GT map samples are illustrated in Figure 4. For evaluation of the generalization capability and robustness of the classifiers, the dataset was randomly split into training set (892 samples) and validation set (200 samples) for training and validation, respectively.

## 3. Methodology

**Figure 5:**
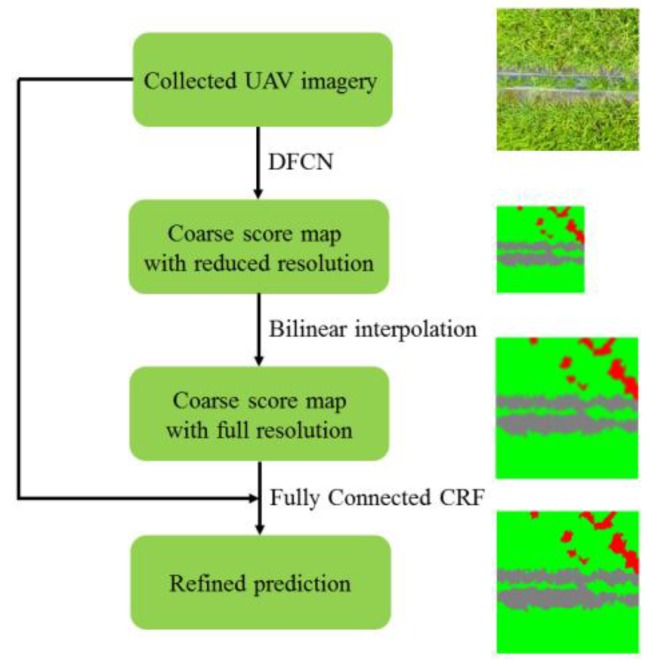
The work-flow of our methodology.

Following the idea of [22], we design a semantic labeling network for weed mapping. The workflow is shown in Figure 5. Firstly, the collected UAV imagery was imported into a Deep Fully Convolutional Network (DFCN), resulting in a coarse score map with reduced resolution. Secondly, the bilinear interpolation was applied to upsample the score map into full resolution. Lastly, the UAV imagery and upsampled score map were exported to a fully connected CRF to further refine the spatial details.

### 3.1. Deep Fully Convolutional Network

The target of our research is to output an accurate weed distribution map, which belong to a semantic labeling task. In recent years, Deep Fully Convolutional Network (DFCN) has been proven effective for semantic labeling in computer vision [13,22] as well as remote sensing [23,24,25] applications. The DFCN can output a dense class map for an input image, making it a potential approach to perform weed mapping tasks of UAV imagery.

#### 3.1.1. Network Architecture

DFCN is a modified version of Deep Convolutional Neural Network (DCNN). In general, traditional DCNNs are composed of a few convolutional layers, pooling layers and fully connected layers [12]. By transforming all the fully connected layers in convolutional forms, a DCNN can be converted into a DFCN, which will output a dense prediction map for the input image [13]. We began our work by adapting proven classification architectures in fully convolutional fashion. In our work, the ResNet [26] and VGG-16 [27] net were considered as the baseline classification architectures.

ResNet was proposed by He et al. [26] in 2015, and won the championship in the ILSVRC15. Compared with prior state-of-art classification architectures [27,28,29], ResNet applied residual learning framework to the plain convolutional network, which well addressed the degradation problem of deep network. It was proven by He et al. [26] that the 152-layer ResNet outperformed others (34-layer, 50-layer, and 101-layer) in terms of accuracy. However, the size of our imagery (1000 × 1000) is larger than that in the ImageNet (224 × 224), which may cause GPU exhaustion with 152-layer network, so the 101-layer ResNet was chosen to be the baseline architecture.

To fit the weed mapping task, the ResNet should be adapted in a fully convolutional fashion. The architectures of ResNet before and after adaption were shown in Table 2 (in the column of layer type, the architecture is shown in blocks, multiplied with the number of blocks). In the baseline architecture of ResNet, downsampling is performed by *conv1*, *conv2_1*, *conv3_1*, *conv4_1*, and *conv5_1* with a stride of 2, resulting in 1/32 downsampling feature maps (*conv5_3*). However, upsampling the feature maps to full resolution needed a 32 pixel stride, limiting the spatial precision of the output. Thus, in the modified architecture, the strides of *conv4_1* and *conv5_1* were set to 1, resulting in 1/8 downsampling feature maps. This change decreases the upsampling factor from 32 times to 8 times, improving the precision of details in the upsampled output. After that, the fully connected layer (*fc6*) was discarded and replaced with a 3 × 3 convolution layer with dimension 3 to predict scores for the whole classes (others, rice, and weeds). The final output of the ResNet was a coarse score map with reduced resolution (size 125 × 125), and was upsampled to full resolution (size 1000 × 1000) using a simple bilinear interpolation. Compared with the deconvolutional approach adopted in [13], bilinear interpolation upsamples a signal without requiring learning any parameters, leading to faster training in practice.

Besides ResNet, VGG-16 net was also considered in this study. VGG-net was the runner-up in the ImageNet ILSVRC-2014, and secured the 1th and 2th places in the localization and classification tasks [27]. As a classification network, VGG-net was popular to be the baseline architecture of the semantic labeling approaches [13,30]. In the baseline architecture of VGG-net, very small (3 × 3) convolution filters were used, and the depth of the network was pushed to 16–19 layers, as shown in Table 3 (In the column of layer type, the architecture is shown in blocks, multiplied with the number of blocks). In our work, the VGG-16 net was adapted to fit our task. Similar to ResNet, the strides of the pool4 and pool5 were set to 1, reducing the degree of signal downsampling (from 1/32 to 1/8). After that, the fully connected layer (*fc6*) was discarded and replaced with a 3 × 3 convolution layer with dimension 3. The output of the modified VGG-16 net was a coarse score map (size 125 × 125), and upsampled to full resolution through a simple bilinear interpolation, same as the ResNet.

#### 3.1.2. Transfer Learning

DCNNs have shown astounding results in remote sensing applications [31,32]. However, with the limited data we had, the training of DCNNs will dramatically overfit the training data. In this work, transfer learning was applied to address this problem. The ImageNet pre-trained models (ResNet, VGG-16 net) were adapted to our task, and their representations were transfered to our dataset by fine-tuning technique.

#### 3.1.3. Atrous Convolution

**Figure 6:**
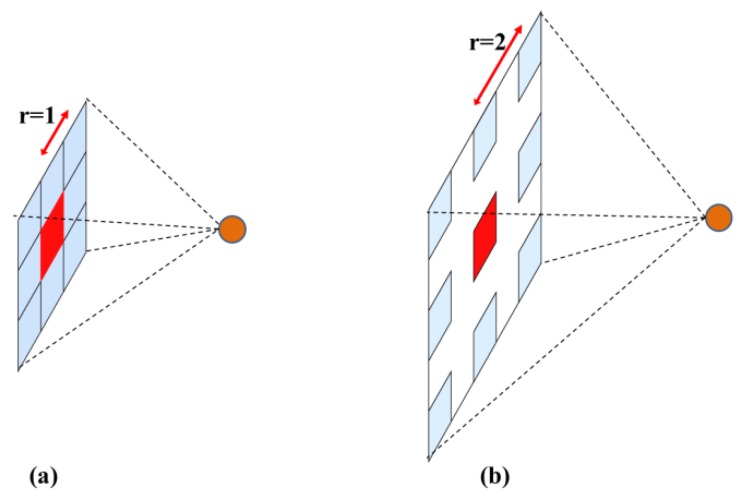
Illustration of two types of convolution. (**a**) Standard convolution. (**b**) Atrous convolution.

Atrous convolution, proposed by Chen et al. [33], was applied to DCNNs for generating dense feature maps. Since the feature maps were computed in 2-D forms, the application of atrous convolution in 2-D situations will be considered in the following. Assuming the input x as a 2-D signal, and the filter w (size K × K) as a 2-D matrix, then the standard convolution of x and w can be defined as:

(1) $$y\left[i,j\right]=\sum_{k=1}^{K}\sum_{l=1}^{K}x\left[i+k,j+l\right]w\left[k,l\right]$$

and the atrous convolution of x and w can be described in the following:

(2) $$y\left[i,j\right]=\sum_{k=1}^{K}\sum_{l=1}^{K}x\left[i+r\cdot k,j+r\cdot l\right]w\left[k,l\right]$$

where r denotes the parameter rate corresponding to the stride. From the Formulas (1) and (2), it can be seen that standard convolution is a special case of atrous convolution with rate = 1. In a 2-D case, the operation of standard convolution and atrous convolution with rate = 2 are illustrated in Figure 6.

In the implementation of the algorithm, the atrous convolution with rate = r inserts r−1 zeros between two adjacent filter values, extending the field of view from K × K to K_1_ × K_1_, where

(3) $$K_{1}=\left(K-1\right)\times \left(r-1\right)+K$$

However, during the computing process, only the nonzero filter values needs to be taken into account. Thus, atrous convolution extends the field of view of filters without extra parameters or computations.

In the setup of network architecture (Section 3.1.1), the last two downsampling operations were removed to increase the spatial resolution of feature maps. However, the field of view of the filters was reduced (i.e., In the architecture of ResNet, the field of view of *conv4_x* layers was reduced by 1/2 × 1/2, and the field of view of *conv5_x* layers was reduced by 1/4 × 1/4), significantly weakening the invariance (to small shifts and distortions of previous layers) created by downsampling. In this work, atrous convolution was used to recover the field of view of the filters. Correspondingly, in the modified architecture of ResNet, the standard convolutional layers of *conv4_x* were replaced with atrous convolution with rate = 2, and the standard convolutional layers of *conv5_x* were replaced with atrous convolution of rate = 4. Similar changes were applied in the modified architecture of VGG-16 net.

#### 3.1.4. Multi-Scale Processing

**Figure 7:**
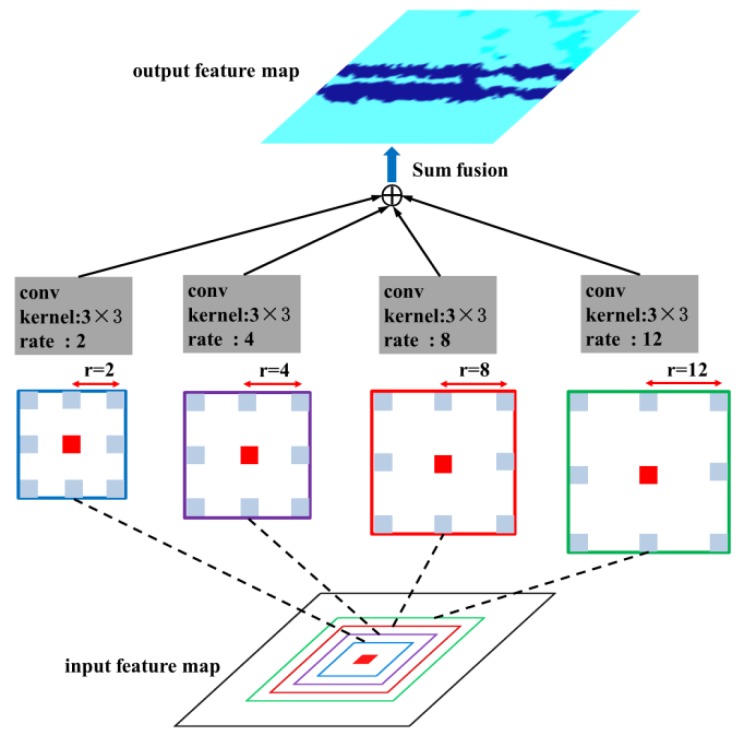
Illustration of multi-scale processing.

In this work, the multi-scale processing simultaneously employs several braches of atrous convolutional layers to a feature map, which may improve the DCNN’s capability to capture objects at different scales. In this scheme, the features are computed at different scales and fused to generate the output. The multiple atrous convolutional layers in multi-scale processing can be implemented in parallel, which significantly improve the efficiency during the network inference. As the setup of ASPP-S in [22], four branches of atrous convolution (r = {2, 4, 8, 12}) was employed in the *fc6* layer (Table 2), which is shown in Figure 7.

### 3.2. Post Processing

The repeated max-pooling and downsampling (‘striding’) in DCNNs significantly improves the invariance to small shifts and reduce the GPU memory involved in network inference. However, these operations cause loss in spatial precision, and generally result in excessive smoothing of spatial details. In this work, fully connected conditional random field (CRF) was employed to refine the spatial details. Fully connected CRF considers each pixel as a CRF node, and the image forms a graph on all nodes. The output labeling $\hat{x}$ of random field is determined by a distribution probability P(x). The distribution probability is related to an energy function E(x), which is designed to refine the spatial precision, as shown in the following:

(4) $$\hat{x}=\begin{array}{c} \mathrm{argmax}\\ x \end{array}\;P\left(x\right)$$

(5) P(x)=exp(−E(x))

(6) $$E\left(x\right)=\sum_{a}\theta_{a}\left(x_{a}\right)+\sum_{a}\sum_{b<a}\theta_{a,b}\left(x_{a},x_{b}\right)$$

where $\theta_{a}\left(x_{a}\right)$ and $\theta_{a,b}\left(x_{a},x_{b}\right)$ represent the unary and pairwise potential, respectively. The unary potential $\theta_{a}\left(x_{a}\right)$ is defined as:

(7) $$\;\theta_{a}\left(x_{a}\right)=-\log\left(P\left(x_{a}\right)\right)$$

where $P\left(x_{a}\right)$ is the probability of the pixel (at location a) exported by a DFCN. The pairwise potential $\theta_{a,b}\left(x_{a},x_{b}\right)$ is defined using the combination of two Gaussian kernels, as shown in the following:

(8) $$\theta_{a,b}\left(x_{a},x_{b}\right)=\mu \left(x_{a},x_{b}\right)\left[w_{1}\exp\left(-\frac{\|p_{a}-p_{b}\|{}^{2}}{2\sigma_{\alpha }^{2}}-\frac{\|I_{a}-I_{b}\|{}^{2}}{2\sigma_{\beta }^{2}}\right)+{\;w}_{2}\exp\left(-\frac{\|p_{a}-p_{b}\|{}^{2}}{2\sigma_{\gamma }^{2}}\right)\right]$$

The first kernel depends on the feature spaces of colors and positions, which was inspired that adjacent pixels with similar colors are likely to have the same type. The second kernel depends only on the feature space of positions, which intends to remove the noise from the output probabilities. The parameters $\sigma_{\alpha }$, $\sigma_{\beta }$, and $\sigma_{\gamma }$ control the shape of the Gaussian kernels. The value of compatibility label $\mu \left(x_{a},x_{b}\right)$ is defined as:

(9) $$\mu \left(x_{a},x_{b}\right)=\{\begin{array}{c} 1,\;x_{a}=x_{b}\\ 0,\;x_{a}\neq x_{b} \end{array}$$

**Figure 8:**
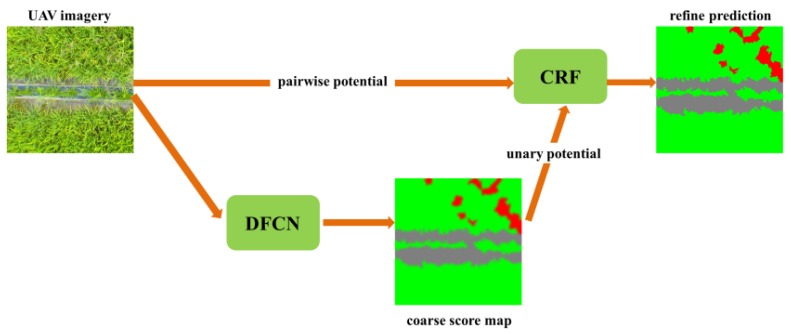
Illustration of the fully connected CRF.

The compatibility label introduces a penalty for adjacent similar pixels with different classes, which significantly improve the precision in spatial details especially along the boundaries, as shown in Figure 8.

### 3.3. Method Comparisons

#### 3.3.1. Pixel-Based-SVM

Following the idea of Alexandridis et al. [8], we performed a per-pixel classification over the input image using the discriminative power of SVM. Different from the model in [8], we used the C-SVC as the model type instead of One-Class-SVM, since there were three target classes (rice, weeds, and others) in our study. The three spectral bands (Red, Green, and Blue) were selected as inputs, and the corresponding classes were set as outputs.

#### 3.3.2. FCN-8s

**Figure 9:**
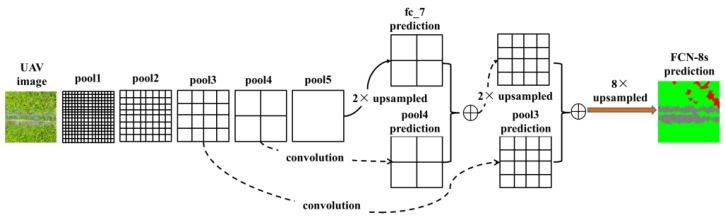
Illustration of skip architecture for FCN-8s. Only the pooling and prediction layers are shown, and other types of layers are ignored in this figure.

In the previous work of our team [16], it was proven that FCN-8s was effective in the weed mapping task of UAV imagery. Thus, in this study, we also compare our algorithm with the FCN-8s method. For FCN-8s, the setup of the network proposed in [13] was used. We adapted the ImageNet pre-trained VGG-16 net and transferred their learned representations to our semantic labeling task. Skip architecture was used to improve the accuracy, as shown in Figure 9. From Figure 9, it can be seen that a 2 ×upsampling operation was conducted to the last convolution layer (resulting in a score map fc_7 prediction), and a 1 × 1 convolutional layer was appended to the pool3 and pool4 (resulting in predictions from pool3 and pool4). The predictions from fc_7 and pool3 were fused with summation, and the result was later fused with the prediction from pool4. Finally, the fused result was upsampled to full resolution, building the final output of FCN-8s.

Although our current work (Figure 5) shared the same fully convolutional framework with previous FCN-8s (Figure 9), there were several new features added to the current approach: (1) residual learning was adopted to address the delegation problem of deep network; (2) atrous convolution was used to reduce the resolution downsampling, thus the skip architecture in FCN-8s was discarded, resulting in a simplified architecture; (3) simple bilinear interpolation was applied for signal upsampling; unlike deconvolutional operation in FCN-8s, parameters in bilinear interpolation does not require optimization, which may significantly accelerate the training process; (4) the fully connected CRF was used as post processing stage, which was not used in FCN-8s. All these newly added features built a significantly improved architecture, leading to better performance in terms of accuracy, which will be shown in Section 4.3.

## 4. Results and Discussions

In the following, the experiments and comparisons will be presented to evaluate our semantic labeling approach. From Section 2.2.2, it can be seen that our dataset was split into training set (892 samples) and validation set (200 samples). All the models were trained on the training set, and the results were reported on the evaluation on the validation set. All the experiments were conducted on a computer with an Intel i7-7700 processor clocked at 3.6G Hz and a NVIDIA GeForce GTX 1080 Ti graphic device.

The mean intersection over union (mean IU), overall accuracy, and Kappa coefficient were employed as the metrics for the experiments. The mean IU counts the mislabeled pixels, which is now the default standard for most semantic labeling competitions (i.e., PASCAL VOC). The Kappa coefficient is obtained by calculating from the confusion matrix, which is a measure that has been used in a variety of applications including semantic labeling tasks.

### 4.1. Deep Fully Convolutional Network

In the experiments of DFCN, (1) the comparison on the performance of different baseline CNN architecture (ResNet-101 and VGG-16 net) was presented; (2) the impact of transfer learning on network training was evaluated; (3) the performance of multi-scale processing was tested. All the experiments in this section were conducted to seek for the optimal network architecture for the weed mapping task.

#### 4.1.1. Network Architecture

In this section, we adapted two proven classification architectures (ResNet-101 and VGG-16 net) into fully convolutional fashion and transfer their learning representations from ImageNet to our weed mapping task (Section 3.1.1). The atrous convolution was applied to the classification network for dense feature extraction (Section 3.1.3). Follow the setup for *fc6* layer in [22], we used the setting of ASPP-L (r = {6, 12, 18, 24}) to enhance the DFCN’s capability to capture objects at different scales. Different from [22], the 1 × 1 convolutional layers of fc_7 and fc_8 were removed, and good performance was observed.

The comparison of performance of ResNet-101 and VGG-16 net was listed in Table 4. From Table 4, it can be seen that the ResNet based DFCN outperforms the VGG-16 net in all terms of metrics. We owed this to the residual learning framework and the increased depth of ResNet. With residual learning, the ResNet enjoyed accuracy gains with increased depth of the network. For the experimental results in this section, the ResNet-101 was chosen as our baseline architecture in the following experiments.

#### 4.1.2. Transfer Learning

**Figure 10:**
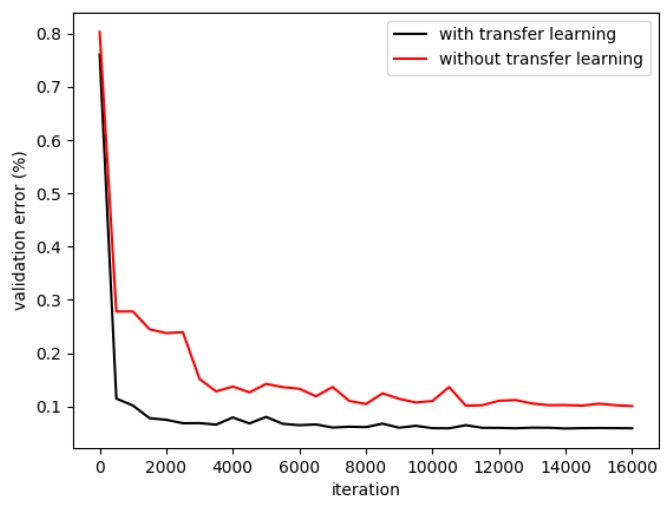
The training process with and without transfer learning.

In this section, two strategies were applied to train the DFCN: (1) the ImageNet pre-trained ResNet was transferred to our dataset by fine-tuning; (2) the ResNet was adapted to our task and trained from scratch. Same as the Section 4.1.1, ASPP-L (r = {6, 12, 18, 24}) was used as the setting for the *fc6* layer of ResNet. The training process was illustrated in Figure 10, and the experimental results were shown in Table 5. From Figure 10 and Table 5, it can be seen that transfer learning significantly accelerates the training process and improves the prediction accuracy. One possible reason for this result was that, without transfer learning, the deep network overfit the limited training data, and this problem was well addressed by the transfer learning method.

#### 4.1.3. Multi-Scale Processing

As described in Section 3.1.4, several branches of atrous convolutional layers with different rates were used in the fc6 layer in order to capture the objects at different scales. Following the setup in [22,33], three settings were adopted in this section: (1) ASPP-12, composed of a single atrous convolutional layer with rate = 12; (2) ASPP-S, composed of four parallel atrous convolutional layers with small rates (r = {2, 4, 8, 12}); (3) ASPP-L, composed of four parallel atrous convolutional layers with large rates (r = {6, 12, 18, 24}). Besides that, the performance of the standard convolution was also evaluated, which was denoted as: (4) ASPP-1, having a single branch with rate = 1.

The experimental results were shown in Table 6. From Table 6, it can be seen that, the performance of the standard convolution version (ASPP-1) outperforms other atrous combinations. One possible reason was that, the resolution of our UVA imagery was constant (0.3 cm) since they were all collected at the same altitude, and the sampling rate of 1 captured the features powerful for discrimination, so adding extra atrous convolutional layers conversely cause the accuracy decrease.

### 4.2. Post Processsing

From Equation (4), it can be seen that the output of fully connected CRF is determined by the probability distribution P(x). However, the high computational cost is the bottleneck for the naïve implementation. Instead, the mean field approximation was used to compute another distribution Q(x) which minimizes the K-L divergence with P(x). The refinement process of CRF generally employs several iterations of mean field approximations, and the number of iterations was set to 10 in our experiments. The value of $w_{2}$ and $\sigma_{\gamma }$ were both set to 1, which was the default setup of [34]. To seek for the optimal value of $w_{1}$, $\sigma_{\alpha }$ and $\sigma_{\beta }$, the grid search strategy was applied on the training set, and this process took around 36 h.

**Figure 11:**
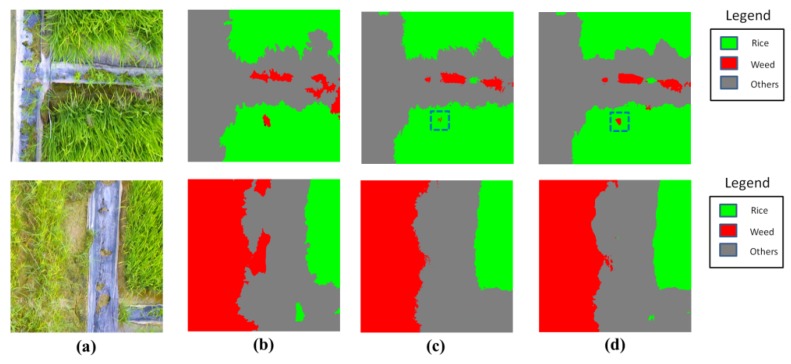
The classification results obtained by ASPP-1 before and after CRFs. (**a**) aerial images; (**b**) corresponding GT labels; (**c**) output by ASPP-1 before CRFs; (**d**) output by ASPP-1 after CRFs.

Two samples on the CRF refinement are given in Figure 11, and the comparisons of quantitative statistics were presented in Table 7. From Figure 11, it can be seen that the output after CRFs has a more clear boundaries, compared to that before CRFs. Especially for the small weed patches (in blue dashed lines), the output after CRFs better delineate the borders. From Table 7, it is obvious that the CRF approach consistently boosts the performance for all models in all metrics. Though the increased margin of accuracy was not significant, the CRF approach can well address the problem of boundary blurring caused by the upsampling operation of our model. This improvement is especially important for the detection of small weed patches.

### 4.3. Comparison with Other Methods

For the setup of Pixel-based-SVM, the Radial Basis Function (RBF) was chosen as the kernel function, and the C-Support Vector Classification (C-SVC) was selected as the objective function. The best penalty factor C was chosen by using grid search strategy. For FCN-8s, the default configuration of [16] was applied in our experiment. For our approach, the architectures of ASPP-1 (with and without CRFs) were adopted based on the experimental results in Table 7.

**Figure 12:**
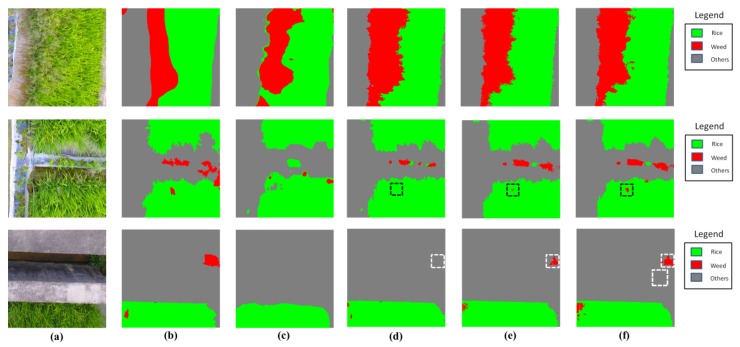
The classification results obtained by methods in comparison. (**a**) aerial images; (**b**) corresponding GT labels; (**c**) output by Pixel-based-SVM; (**d**) output by FCN-8s; (**e**) output by ASPP-1 without CRFs; (**f**) output by ASPP-1 with CRFs.

Figure 12 illustrates the classification results by the involved methods. It can be seen from Figure 12 that, semantic labeling approaches (FCN-8s and ours) significantly outperformed pixel-based-SVM in prediction accuracy. In the pixel-based-SVM approach, each pixel was input to the model independently, which ignored the correlations between pixels and resulted in pool performance in accuracy. With regard to the comparison with FCN-8s, our approach showed significant improvement in detecting small weed patches. From Figure 12, it is obvious that FCN-8s fails to detect the small weed patches distributed inside and outside the rice (in black and white dashed lines, respectively), while our model variants (ASPP-1 without and with CRFs) accurately locate the targets. We owed the improvement to the following reasons: (1) the residual learning framework in our network boosts the accuracy with increased depth; (2) the atrous convolution extends the field of view of convolutional filters, improving the network capability to capture the object at small scale, which leaded to a better performance in detection of small weed patches.

Table 8 lists the experimental results in terms of accuracy and efficiency. From Table 8, it can be seen that semantic labeling approaches (FCN-8s and ours) significantly outperformed the Pixel-based-SVM in terms of accuracy and efficiency. In the pixel-based approach, the labeling processing is performed pixel by pixel, which introduces abundant computation and slows down the inference speed. Compared with FCN-8s, our ASPP-1 model achieved better accuracy at an acceptable speed. However, the employment of CRF significantly slows down the inference, and the extra time for this step is up to 2.6255 s per image. One important reason for this is that the CRF algorithm is implemented in a single CPU thread, which is much slower than those driven by parallelism mechanism or hardware acceleration. Nevertheless, our approach obtained an acceptable accuracy even without the CRF. Thus, in some applications asking for fast image processing, the post processing of CRF can be discarded in order to speed up the network inference.

Table 9 lists the confusion matrix of FCN-8s and our approach. In order to reduce the inference time, the ASPP-1 with CRF was not considered in this case, since the ASPP-1 without CRF has already achieved a competitive performance for weed mapping. From Table 9, it can be seen that our approach obtained higher weed detection rate than Pixel-based-SVM and FCN-8s, which may provide more trustable decision support information for precise herbicide treatment in real applications.

## 5. Conclusions

In this work, high resolution UAV images were collected on a rice field. A semantic labeling approach was applied to automatically generate accurate weed distribution maps. An ImageNet pre-trained ResNet was employed and adapted to our weed mapping task in a fully convolutional fashion, and the learned representations of ResNet were transferred to our dataset using fine-tuning. Atrous convolution was used to extend the field of view of convolutional filters; the performance of multi-scale processing was evaluated; and a fully connected CRF was applied to improve the spatial precision. Our approach was compared with the Pixel-based-SVM and the classical FCN-8s. Comparison results showed that our approach achieved higher accuracy than Pixel-based-SVM and FCN-8s. Especially for the detection of small weed patches, our approach significantly outperformed other methods. All the experimental results demonstrated that our approach has potential to generate accurate weed distribution maps to provide decision support information for herbicide treatment applications. However, the increased complexity of the network leads to a decrease in inference speed, which may limit the applications of our approach. Especially in the post-processing stage, much time is needed to carry out the CRF process. Therefore, one of the future directions is to simplify the network architecture and accelerate the inference process, which will remain as the future work of our study.

## Figures and Tables

**Table 1:** Technical characteristics of the UAV platform.

Parameters	Specifications
Weight (battery included)	1380 g
Max Flight Time	28 min
Max speed	20 m/s
Typical operating altitude	10–300 m
Resolution	4000 × 3000 pixels
Len	35 mm
Typical spatial resolution (at 6 m altitude)	0.3 cm

**Table 2:** Architectures of ResNet before and after adaption.

	Baseline Architecture (before Adaption)		Modified Architecture (after Adaption)	
Layer Name	Size of Output	Layer Type	Size of Output	Layer Type
*conv1*	500 × 500	7 × 7, 64 (stride 2)	500 × 500	7 × 7, 64 (stride 2)
		max-pooling (stride 2)		max pooling (stride 2)
*conv2_x*	250 × 250	$\left[\begin{array}{c} 1\times 1,\;64\\ \begin{array}{c} 3\times 3,\;64\\ 1\times 1,\;256 \end{array} \end{array}\right]\times 3$	250 × 250	$\left[\begin{array}{c} 1\times 1,\;64\\ \begin{array}{c} 3\times 3,\;64\\ 1\times 1,\;256 \end{array} \end{array}\right]\times 3$
*conv3_x*	125 × 125	$\left[\begin{array}{c} 1\times 1,\;128\\ \begin{array}{c} 3\times 3,\;128\\ 1\times 1,\;512 \end{array} \end{array}\right]\times 4$	125 × 125	$\left[\begin{array}{c} 1\times 1,\;128\\ \begin{array}{c} 3\times 3,\;128\\ 1\times 1,\;512 \end{array} \end{array}\right]\times 4$
*conv4_x*	64 × 64	$\left[\begin{array}{c} 1\times 1,\;256\\ \begin{array}{c} 3\times 3,\;256\\ 1\times 1,\;1024 \end{array} \end{array}\right]\times 23$	125 × 125	$\left[\begin{array}{c} 1\times 1,\;256\\ \begin{array}{c} 3\times 3,\;256\\ 1\times 1,\;1024 \end{array} \end{array}\right]\times 23$
*conv5_x*	32 × 32	$\left[\begin{array}{c} 1\times 1,\;512\\ \begin{array}{c} 3\times 3,\;512\\ 1\times 1,\;2048 \end{array} \end{array}\right]\times 3$	125 × 125	$\left[\begin{array}{c} 1\times 1,\;512\\ \begin{array}{c} 3\times 3,\;512\\ 1\times 1,\;2048 \end{array} \end{array}\right]\times 3$
*fc6*	1 × 1	1000-d fc, softmax	125 × 125	3 × 3, 3

**Table 3:** Architectures of VGG-16 net before and after adaption.

	Baseline Architecture (Before Adaption)		Modified Architecture (After Adaption)	
Layer Name	Size of Output	Layer Type	Size of Output	Layer Type
*conv1*	1000 × 1000	[3 × 3, 64] × 2	1000 × 1000	[3 × 3, 64] × 2
*pool1*	500 × 500	max-pooling (stride 2)	500 × 500	max-pooling (stride 2)
*conv2_x*	500 × 500	[3 × 3, 128] × 2	500 × 500	[3 × 3, 128] × 2
*pool2*	250 × 250	max-pooling (stride 2)	250 × 250	max-pooling (stride 2)
*conv3_x*	250 × 250	[3 × 3, 256] × 3	250 × 250	[3 × 3, 256] × 3
*pool3*	125 × 125	max-pooling (stride 2)	125 × 125	max-pooling (stride 2)
*conv4_x*	125 × 125	[3 × 3, 512] × 3	125 × 125	[3 × 3, 512] × 3
*pool4*	64 × 64	max-pooling (stride 2)	125 × 125	max-pooling (stride 1)
*conv5_x*	64 × 64	[3 × 3, 512] × 3	125 × 125	[3 × 3, 512] × 3
*pool5*	32 × 32	max-pooling (stride 2)	125 × 125	max-pooling (stride 1)
*fc6*	1 × 1	1000-d fc, softmax	125 × 125	3 × 3, 3

**Table 4:** Experimental results of different baseline architectures.

Approach	Mean IU	Overall Accuracy	Kappa
ResNet-101	0.7668	0.9409	0.9076
VGG-16 net	0.7478	0.9350	0.8979

**Table 5:** Experimental results of transfer learning.

Approach	Mean IU	Overall Accuracy	Kappa
ResNet-101 with transfer learning	0.7668	0.9409	0.9076
ResNet-101 without transfer learning	0.6959	0.8995	0.8417

**Table 6:** Experimental results of ASPP.

Approach	Mean IU	Overall Accuracy	Kappa
ASPP-12	0.7660	0.9395	0.9054
ASPP-S	0.7703	0.9397	0.9059
ASPP-L	0.7668	0.9409	0.9076
ASPP-1	0.7721	0.9423	0.9094

**Table 7:** Experimental results of CRF.

Approach	Mean IU	Overall Accuracy	Kappa
ASPP-12 before CRF	0.7660	0.9395	0.9054
ASPP-12 after CRF	0.7690	0.9415	0.9084
ASPP-S before CRF	0.7703	0.9397	0.9059
ASPP-S after CRF	0.7731	0.9417	0.9088
ASPP-L before CRF	0.7668	0.9409	0.9076
ASPP-L after CRF	0.7674	0.9433	0.9112
ASPP-1 before CRF	0.7721	0.9423	0.9094
ASPP-1 after CRF	0.7751	0.9445	0.9128

**Table 8:** Classification results of FCN-8s and our approach. The metrics of speed is measured in seconds by the inference time of a single image.

Approach	Mean IU	Overall Accuracy	Kappa	Speed
Pixel-based-SVM	0.6549	0.8513	0.6451	233.7187 s
FCN-8s	0.7478	0.9350	0.8979	0.1406 s
ASPP-1 without CRF	0.7721	0.9423	0.9094	0.2916 s
ASPP-1 with CRF	0.7751	0.9445	0.9128	2.9171 s

**Table 9:** Confusion matrix of FCN-8s and our approach.

Approach	GT/Predicted Class	Others	Rice	Weeds
Pixel-based-SVM	others	**0.898**	0.054	0.048
	rice	0.037	**0.865**	0.098
	weeds	0.141	0.128	**0.731**
FCN-8s	others	**0.940**	0.050	0.010
	rice	0.027	**0.956**	0.017
	weeds	0.063	0.054	**0.883**
ASPP-1 before CRF	others	**0.950**	0.034	0.016
	rice	0.039	**0.944**	0.017
	weeds	0.050	0.025	**0.925**
